# Altered Actin Dynamics in Cell Migration of GNE Mutant Cells

**DOI:** 10.3389/fcell.2021.603742

**Published:** 2021-03-18

**Authors:** Shamulailatpam Shreedarshanee Devi, Rashmi Yadav, Ranjana Arya

**Affiliations:** School of Biotechnology, Jawaharlal Nehru University, New Delhi, India

**Keywords:** sialylation, actin dynamics, RhoA, cofilin, cell migration, GNE myopathy

## Abstract

Cell migration is an essential cellular process that requires coordination of cytoskeletal dynamics, reorganization, and signal transduction. The actin cytoskeleton is central in maintaining the cellular structure as well as regulating the mechanisms of cell motility. Glycosylation, particularly sialylation of cell surface proteins like integrins, regulates signal transduction from the extracellular matrix to the cytoskeletal network. The activation of integrin by extracellular cues leads to recruitment of different focal adhesion complex proteins (Src, FAK, paxillin, etc.) and activates the signal including Rho GTPases for the regulation of actin assembly and disassembly. During cell migration, the assembly and disassembly of actin filament provides the essential force for the cell to move. Abnormal sialylation can lead to actin signaling dysfunction leading to aberrant cell migration, one of the main characteristics of cancer and myopathies. In the present study, we have reported altered F-actin to G-actin ratios in GNE mutated cells. These cells exhibit pathologically relevant mutations of GNE (UDP N-acetylneuraminic 2-epimerase/N-acetylmannosamine kinase), a key sialic acid biosynthetic enzyme. It was found that GNE neither affects the actin polymerization nor binds directly to actin. However, mutation in GNE resulted in increased binding of α-actinin to actin filaments. Further, through confocal imaging, GNE was found to be localized in focal adhesion complex along with paxillin. We further elucidated that mutation in GNE resulted in upregulation of RhoA protein and Cofilin activity is downregulated, which could be rescued with Rhosin and chlorogenic acid, respectively. Lastly, mutant in GNE reduced cell migration as implicated from wound healing assay. Our study indicates that molecules altering Cofilin function could significantly revert the cell migration defect due to GNE mutation in sialic acid-deficient cells. We propose cytoskeletal proteins to be alternate drug targets for disorders associated with GNE such as GNE myopathy.

## Introduction

Cell adhesion and migration is a key function of cells, mediated by complex fibrous network of protein filaments in the cytoplasm creating what is known as cytoskeleton. The protein fibers comprise of microtubules, microfilaments, and intermediate filaments (Schaks et al., 2019). In particular, actin microfilament plays a vital role in maintaining cell morphology, motility, trafficking, endocytosis, cytokinesis, and apoptosis (Deng et al., 2018). The function of actin relies primarily on the dynamic assembly and disassembly of actin filaments into monomeric G (globular) actin and filamentous F (filamentous) actin forms. The monomeric or globular forms of actins assemble to form F-actin filaments of 7 nm along with various actin-binding proteins (Ananthakrishnan and Ehrlicher, 2007; Lee and Dominguez, 2010). The kinetics of actin filament is limited by a nucleation step that involves formation of actin dimers and trimers. The formation of actin trimer triggers elongation of actin monomers into a right-handed helical polymer with two distinct ends—barbed end and pointed end. Active polymerization occurs at the barbed end while depolymerization occurs at the pointed end. The process is tightly regulated by various molecular machines such as Arp 2/3, formins, WASP (Wiskott–Aldrich syndrome protein) superfamily, and Rho GTPases (Bassagañas et al., 2014a; Onishi et al., 2018; Kang and Woo, 2019). Besides actin polymerization, the turnover rate of filaments is tightly regulated by depolymerizing factors such as Cofilin and capping proteins (Sousa-Squiavinato et al., 2019). Further, a large number of F-actin binding and bundling proteins determine fate, longevity, and function of actin filaments (Biron and Moses, 2004).

Polymerization of G-actin monomer to F-actin is critical for cell migration and is regulated by several signaling pathways. Integrin, one of the important transmembrane cell receptors, interacts with ECM and is involved in actin dynamic regulation. Activated integrins control downstream signaling pathway through focal adhesion kinase (FAK), Src, Paxillin, α-actinin, kindlin, talin, and many more (Steffen et al., 2017). FAK activates Rho/Rac/CDC42 that control actin branching and polymerization via WASP and Arp 2/3 proteins (Chen et al., 2011). RhoA is also reported to be active in leading-edge, membrane ruffling and lamellae formation during cell migration (O'Connor and Chen, 2013). Alternately, RhoA also regulates the formation of stress fiber that reduces cell motility (Huveneers and Danen, 2009). RhoA regulates the actin dynamics through ROCK kinase that further activates the LIM Kinase and myosin light chain phosphatase (MLCP) (Swärd et al., 2003; O'Connor and Chen, 2013). While phosphorylation of MLCP by ROCK results in decreased phosphatase activity of MLCP, the activated LIMK phosphorylates Cofilin at Ser3, thereby deactivating Cofilin. Cofilin is a key actin-binding protein that performs an essential role in F-actin severing by binding to G-actin and blocking its incorporation into F-actin (Hoffmann et al., 2019). Thus, Cofilin phosphorylation leads to loss of its actin binding, depolymerizing, and severing activity. Cofilin is activated via SSH1 (slingshot homolog 1)-mediated dephosphorylation to induce F-actin severing for creation of new barbed and pointed ends during actin polymerization (Woo et al., 2019).

In addition to various signaling cascades, actin is regulated in the cell through different posttranslational modifications such as acetylation, glycosylation, methylation, ubiquitination, arginylation, SUMOylation, ADP-ribosylation, Met-oxidation, carbonylation, glutathionylation, and S-nitrosylation. Increased O-GlcNAcylation of actin has been reported to lower actin polymerization, eventually lowering the F-actin levels in the cells (Terman and Kashina, 2013; Akimoto et al., 2019). O-linked and N-acetylglucosaminylation was observed for other contractile proteins affecting the cytoskeletal network (Hedou et al., 2007). Among the different glycosylations, the incorporation of sialic acid, a nine-carbon atom and acidic sugar, is important for different cytoskeletal proteins. Alterations in sialylation of cell surface antigens markedly affects cell metastasis and invasion abilities (Häuselmann and Borsig, 2014; Li and Ding, 2019). Overexpression of sialyltransferases leads to increased sialylation of membrane glycoproteins and contribute to metastasis by enhancing motility while inhibition of sialyltransferases blocks invasion (Shaikh et al., 2008). Inhibitors of sialyltransferase suppress FAK/Paxillin signaling and cell metastasis (Chen et al., 2011). Sialylation is important for ligand binding to selectins and blocking of this interaction helps in reducing the metastasis of cancer cells (Natoni et al., 2016). Even sialylated integrins demonstrate greater adhesion and migration on collagen I compared to unsialylated integrin-expressing cells (Seales et al., 2005). Also, hyposialylated integrins in sialic acid-deficient HEK293 cells depicted reduced adhesion (Grover and Arya, 2014). Thus, it is of interest to determine the effect of alteration in glycosylation, particularly sialylation, on cell migration machinery.

The process of sialylation of glycoproteins starts from the biosynthesis of sialic acid from UDP-N-acetylglucosamine. The critical enzyme involved in the biosynthesis of sialic acid is GNE (UDP-N-acetylglucosamine 2-Epimerase/N-acetylmannosamine kinase) (Devi et al., 2018). Mutations in GNE cause two different types of genetic disorder—sialuria and GNE myopathy. Sialuria is a rare genetic disorder caused by mutation in the allosteric site of GNE leading to failure of feedback mechanism of GNE enzyme, resulting in excess excretion of sialic acid in the urine. Mutation in GNE in epimerase or kinase domain results in reduced sialic acid content leading to hyposialylation of different proteins such as integrins, NCAM, and IGF1R (Grover and Arya, 2014; Singh et al., 2018). The pathological significance of GNE mutation leads to a neuromuscular rare genetic disorder, GNE myopathy, that affects skeletal muscle cell function. Sela et al. had reported that different cytoskeletal proteins including α- and γ-actin, myosin light chain, troponin, tropomyosin-3, Zyxin, Vimentin, Nexilin, and radixin were differentially expressed in GNE myopathy muscle samples (Sela et al., 2011). Weidemann et al. had also reported that GNE interacts with CRMP1 (Collapsin response-mediated protein-1), the cytoskeletal regulatory protein (Weidemann et al., 2006). Amsili et al. had shown that GNE directly interacts with α-actinin-1 (Amsili et al., 2008) while Harazi et al. showed that GNE interacts with α-actinin-2 and mutation in GNE led to strengthening the bond between GNE and α-actinin-2 (Harazi et al., 2017). Thus, various studies indicate altered cytoskeletal protein network in the presence of mutant GNE proteins in the cell. Whether mutation in GNE leads to dysregulation in cytoskeletal organization is not clear. Is it hyposialylation that causes alteration in cytoskeletal organization or is it GNE protein that has alternate function needs to be addressed in detail? Further supplementation with sialic acid in GNEM patients slows the disease progression, suggesting that hyposialylation may not be the only cause of the disease. Thus, there is utmost need to understand the pathomechanism at the cellular and molecular level and identify potential therapeutic targets besides sialic acid.

A major limitation in understanding the effect of GNE mutations on cellular functions is the lack of an appropriate cell-based model system. GNE knockout mice were found to be embryonically lethal (Schwarzkopf et al., 2002) and GNE transgenic mice showed variations in the phenotype with renal failure (Sela et al., 2013). Therefore, in our laboratory, a HEK cell-based system with overexpressed pathologically relevant GNE mutations has been used to understand the pathomechanism of disease at the molecular level. In the present study, we aim to decipher the effect of reduced sialylation on actin dynamics via integrin signaling pathway in the HEK293 cell-based model for GNE myopathy (Grover and Arya, 2014; Singh and Arya, 2016; Chanana et al., 2017; Singh et al., 2018). The effect of RhoA and Cofilin is elucidated in sialic acid-deficient cells, and molecules altering their activity have been assessed for restoring the defects. Our study will provide insights into cytoskeletal protein function in GNE mutant cells and offer potential therapeutics for the disorder.

## Materials and Methods

### Cell Lines and Preparation for Whole Cell Lysate

HEK293 cells overexpressing recombinant GNE protein with mutation in the epimerase domain (D207V-GNE) and kinase domain (V603L-GNE) were used in the study. As sialic acid is a component of serum, DCCM (Biological Industries), serum-free media was used to avoid sialic acid uptake from the medium.

Cells were seeded in DMEM media followed by incubation in DCCM media for 24 h. The cells were lysed using 1 ml of RIPA lysis buffer containing 20 mM Tris–HCl (pH 7.5), 1% NP-40 (Sigma Aldrich), 150 mM NaCl, 1 mM EDTA, and protease inhibitor cocktail (Sigma Aldrich). The cell lysates were incubated for 45 min on ice. Cells were collected and centrifuged at 13,000 rpm to remove cell debris. The supernatant was collected, and protein content was estimated using Bradford reagent (Sigma Aldrich).

### Preparation of G-Actin and F-Actin Fraction

Cells were cultured in 100-mm dishes in DCCM media for 24 h. Cells were washed with PBS buffer and lysed with 1 ml of F-actin stabilizing lysis solution containing 50 mM PIPES, pH 6.9, 50 mM NaCl, 5 mM MgCl_2_, 5 mM EGTA, 5% glycerol, 0.1% Triton X-100, 0.1% Tween-20, 0.1% NP-40, 0.1% 2-mercaptoethanol, 1 mM ATP, and protease inhibitor cocktail for 45 min on ice. The cells were scraped and centrifuged at 100,000 × *g* for 1 h. The supernatant containing the G-actin fraction was collected while the pellet was dissolved in 1 ml of chilled water containing 1 mM cytochalasin D and incubated for 1 h. The pellet was centrifuged at 13,000 rpm for 30 min, and the supernatant was collected for F-actin fraction. Equal volumes of G-actin and F-actin fraction were subjected for immunoblot analysis using an anti-β-actin antibody [β-actin (C4), Santacruz Antibodies], and imaging was done by Enhanced Chemiluminescence (ECL) using ChemiDoc Imaging Systems, Bio-Rad. The F/G-actin ratio was determined by densitometry of the immunoblots.

### Fibronectin Stimulation

Cells were grown in DCCM media for 24 h prior to fibronectin stimulation. The cells were trypsinized and subjected to fibronectin (Sigma Aldrich) stimulation for 4 h in 100-mm cell culture dishes at 37°C.

### Confocal Microscopy

Cells were grown in tissue culture plates containing sterile coverslip in DCCM media for 24 h. Cells were then fixed with 3.7% paraformaldehyde and stained with the primary antibody in antibody diluting solution (1% BSA and 0.05% Triton X-100 in 1 × PBS) for 2 h followed by Alexa Fluor-tagged secondary antibody (Molecular Probes) for 45 min. Cells were stained with 1 μg/μl Hoechst nuclear stain for 10 min. Cells were mounted on slides using DABCO (Sigma Aldrich). The images were visualized using an Olympus FluoView FV1000 laser scanning microscope.

### TRITC-Phalloidin Staining

After fixing the cells using 3.7% paraformaldehyde, cells were stained with 1:300 dilution of TRITC-phalloidin (Sigma Aldrich) and 1 μg/μl of Hoechst nuclear stain for 30 min and 10 min, respectively. Images were acquired at 555 nm in Olympus FluoView FV1000 ver1.7. Quantitative analysis was done using Olympus FluoViewFV1000 ver1.7a software and ImageJ software.

### RNA Extraction

RNA samples were extracted from cells seeded in a six-well plate using TRIZOL. Briefly, cells were washed with 1 × PBS and lysed for 5 min with TRIZOL reagent (Bio Basic, Inc., Canada). Two hundred microliters of chloroform was added to the mixture followed by mixing and centrifuged at 12,000 × *g*. The upper aqueous layer was collected, and 500 μl of isopropanol was added followed by mixing the solution. The mixture was centrifuged, and the supernatant was discarded while the collected pellet was washed with 80% ethanol and centrifuged at 12,000 × *g* for 10 min. The pellet was dried in room temperature for and dissolved in 30 μl of RNase-free water supplemented with Ribolock RNase inhibitor (Thermo Scientific).

### cDNA Synthesis

cDNA was synthesized using 10 μg of RNA using Reverse Transcriptase (Thermo Scientific) following the manufacturer's manual. Briefly, the reaction was incubated at 25°C for 5 min, 40°C for 60 min, and 70°C for 10 min in a Thermocycler (Applied Biosystem, USA).

### qRT-PCR Condition for RhoA

Primer 1—CATTTCTGTCCCAACGTGCC; primer 2—TTCCCACGTCTAGCTTGCAG. Denaturation at 95°C for 30 s, annealing at 58°C for 20 s, and extension at 72°C for 30 s for 25 cycles.

### qRT-PCR Condition for Cofilin

Primer 1—TCTCTGATGGTGTCATCAAGGTGTT; Primer 2—ATAGGTTGCATCATAGAGGGCATAG. Denaturation at 95°C for 30 s, annealing at 55°C for 20 s, and extension at 72°C for 30 s for 25 cycles.

### GTPase Assay

HEK293 cells were lysed with GST-FISH buffer [25 mM Tris–Cl, pH 7.2, 150 mM NaCl, 5 mM MgCl_2_, 0.5% NP-40, 5% glycerol, 1 mM PMSF (Sigma Aldrich), and PIC (Protease Inhibitor cocktail)] for 10 min followed by centrifugation at 10,000 rpm for 15 min at 4°C. One milligram of protein lysates was incubated with purified RBD-GST-bound Sepharose beads (GE Healthcare, USA) for 2 h at 4°C with gyration. Incubated beads were collected by centrifugation at 2,500 rpm followed by washing thrice with chilled PBS buffer. The protein-bound beads were dissolved in 40 μl of 4 × SDS loading buffer and boiled for 10 min. After brief centrifugation, the supernatant was subjected to SDS-PAGE analysis and immunoblotting was done with RhoA antibody (Santacruz Antibodies).

### Rhosin Treatment

Cells were grown in DCCM media supplemented with L-glutamine for 24 h followed by treatment of Rhosin 0 μM, 5 μM, 10 μM, 15 μM, and 30 μM for 2 h. The cells were lysed, and proteins were run on SDS-PAGE and Western transferred followed by immunoblotting.

### CGA Treatment

Cells were grown in DCCM media supplemented with L-glutamine for 24 h followed by treatment of chlorogenic acid (CGA) 0 μM, 1 μM, and 5 μM for 24 h. The cells were lysed, and proteins were run on SDS-PAGE and Western transferred followed by immunoblotting.

### Wound Healing Assay

HEK293 cells were counted and 0.5 × 10^5^ cells were seeded in each chamber in IBIDI inserts followed by incubation at 37°C for 24 h in DCCM media. The inserts were removed, and cells were washed with DCCM media. DCCM media (500 μl) was added and images were captured at 20 × magnification in a Zeiss Axio Vert.A1 inverted microscope (Carl Zeiss AG, Germany).

### Antibodies

Primary antibodies for β-actin (1:2000 dilution), RhoA (1:1000), α-actinin-1 (1:1000), Cofilin (1:1000 dilution), c-Myc (1:1000), GAPDH (1:2000), and GNE (1:1000) were purchased from Santa Cruz Biotechnology, USA. HRP-conjugated secondary antibodies were used with dilution of (1:5000).

## Results

### Effect of GNE Mutation on Actin Dynamic Levels

**Figure 1:**
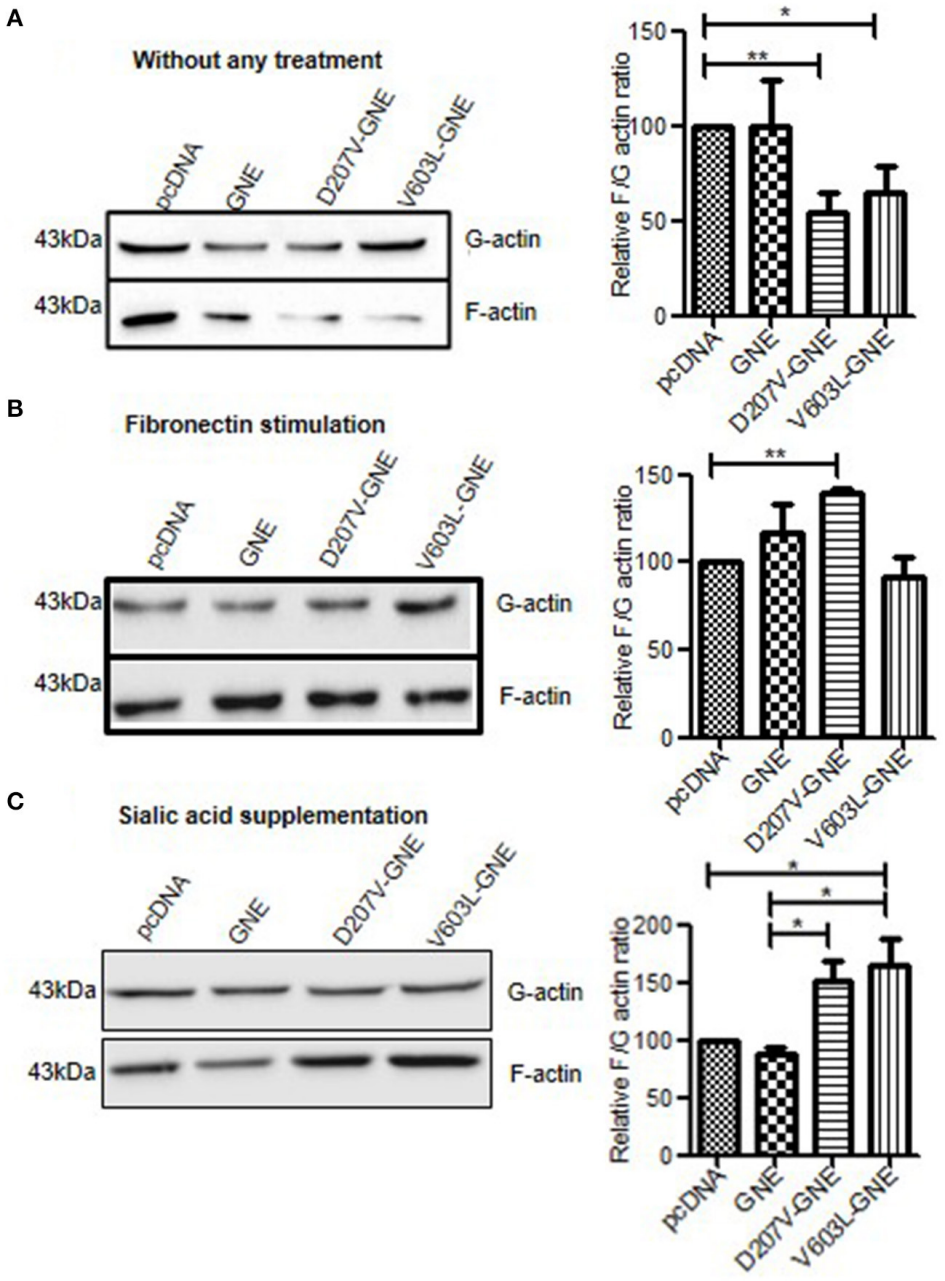
Analysis of F/G-actin ratio in GNE mutant cells. **(A)** GNE mutant cells and control cells were grown in DCCM for 24 h. Cells were lysed in F-actin stabilizing buffer and G-actin fractions were separated and collected as supernatant after centrifugation at 100,000 × *g*. The pellets (F-actin fraction) were dissolved in chilled water containing 1 nM cytochalasin and incubated for 1 h to depolymerize to F-actin followed by centrifugation at 13,000 rpm, and the supernatant was collected as F-actin fraction. Equal volumes of lysate (40 μl) of G-actin and F-actin were loaded on SDS-PAGE followed by immunoblotting with anti-β-actin antibody. Densitometric analysis of the blot was done using ImageJ software and statistical significance of the data was assessed by one-way ANOVA test. *p*-value for * is <0.05 and ** is <0.005 and bar represent SEM, *n* = 3. **(B)** F/G-actin ratio after fibronectin stimulation. Cells were grown in DCCM media for 24 h followed by fibronectin stimulation for 4 h. F/G-actin ratio were analyzed by immunoblotting. **(C)** F/G-actin ratio after sialic acid supplementation. Cells were grown in DCCM media for 24 h followed by supplementation of 5 mM sialic acid for another 24 h. The F/G-actin ratio were analyzed by immunoblotting followed by densitometric analysis by ImageJ software and statistical significance of the data was assessed by one-way ANOVA test. *p*-value for * is <0.01 and the bar represent SEM, *n* = 3.

Polymerization and depolymerization of actin are strictly regulated in cellular processes like cell adhesion and migration. The cells maintain equilibrium between G (globular)-actin and F (filamentous)-actin form with the help of various cytoskeletal and signaling proteins. Whether changes in sialylation levels of the cell affect actin dynamics was addressed in the present study. For this purpose, HEK293 cells overexpressing recombinant GNE mutant proteins (D207V-GNE and V603L-GNE) and exhibiting reduced sialic acid content due to reduced GNE activity were used (Grover and Arya, 2014). D207V and V603L mutations are the most predominant pathologically relevant epimerase and kinase domain. Previous studies on these cells demonstrated that hyposialylated integrin activated downstream signaling in response to fibronectin leading to increased adhesion of GNE-deficient cells. How changes in sialic acid content affect actin dynamic levels in GNE-deficient cells was studied by determining F/G-actin ratio in these cells. G-actin and F-actin fractions were isolated and subjected to immunoblot analysis as described in section Materials and Methods. F/G-actin ratios were determined by densitometric analysis of immunoblots. F/G-actin ratio was reduced by 55% (SEM ±6.2) and 40% (SEM ±5.9) D207V-GNE and V603L-GNE mutant cells, respectively, as compared to wild-type GNE and vector control (Figure 1A). This study suggests that F-actin levels are reduced in sialic acid-deficient cells. However, in response to fibronectin stimulation, F/G-actin ratio increased by three-fold in D207V-GNE and two-fold in V603L-GNE mutant cells, as compared to wild-type GNE and vector control (Figure 1B). These data support our earlier observation that fibronectin stimulation in GNE mutant cells led to β-1 integrin downstream signaling activation, and therefore, F-actin levels are also increased. Further, since mutation in GNE reduces sialic acid content in the cells, the effect of sialic acid supplementation on F/G-actin ratio was studied in GNE mutant protein-overexpressing cells. As shown in Figure 1C, supplementation of cells with 5 mM sialic acid restored the F-actin levels in GNE mutant cells similar to wild-type GNE overexpressing and vector control cells. Our study indicates that in the presence of mutant GNE proteins, F/G-actin dynamic levels are reduced in the cells that may affect overall the cytoskeletal network in the cell.

### Validation of Altered F-Actin Levels in GNE Mutant Cells

**Figure 2:**
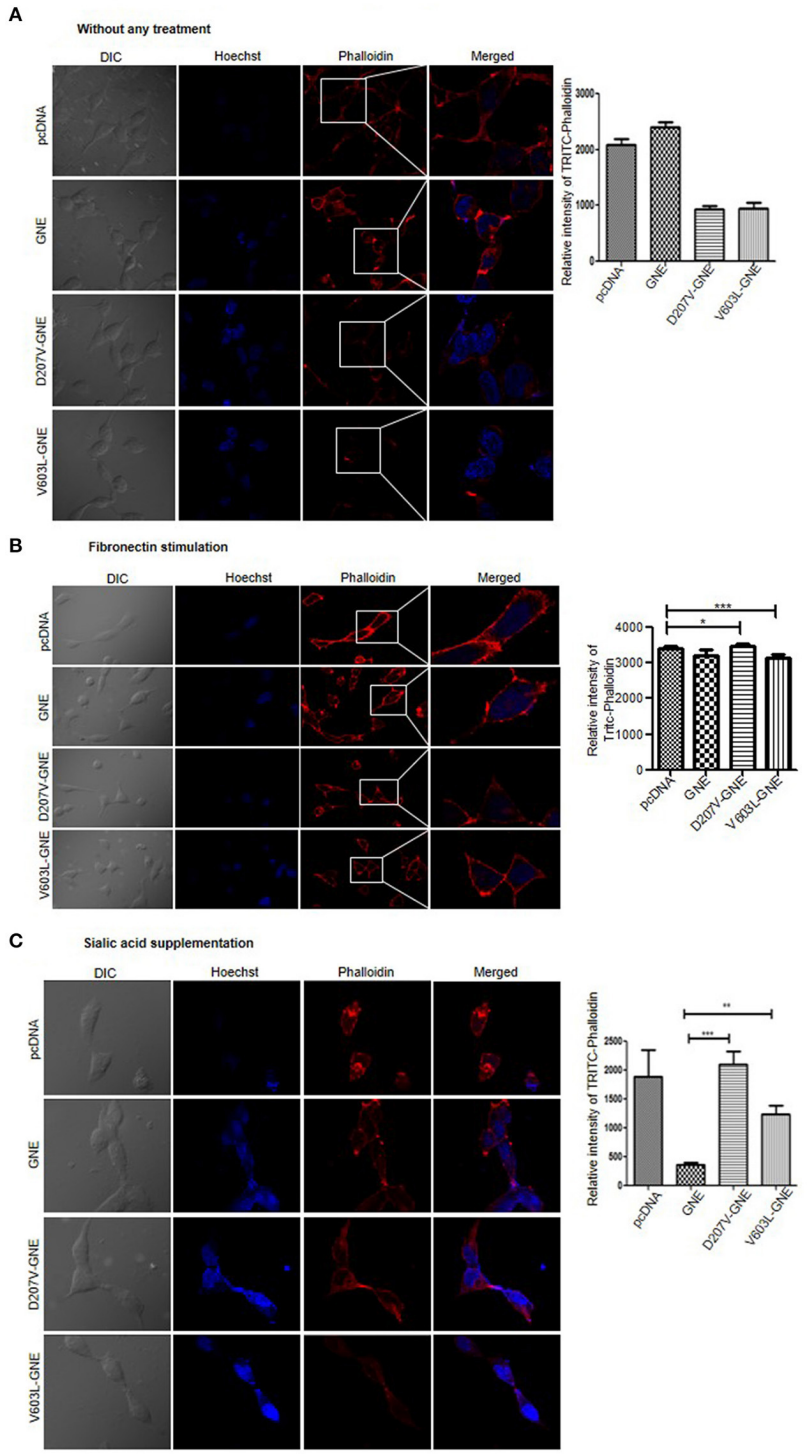
Analysis of F-actin by using Phalloidin. **(A)** Phalloidin, the compound that binds to F-actin selectively, was used to visualize the F-actin through confocal microscopy. Cells were grown in DCCM media for 24 h and fixed using paraformaldehyde. The cells were stained using TRITC-phalloidin and images were captured at 555 nm at 60 × magnification. The graph represents the quantitative analysis of fluorescent intensity of TRITC-phalloidin using FV10-ASW 1.7-Olympus software. **(B)** F-actin staining after fibronectin stimulation. Cells, after growing in DCCM media for 24 h, were stimulated with fibronectin for 4 h followed by TRITC-phalloidin staining and images were captured at 555 nm at 60×. **(C)** F-actin staining after sialic acid supplementation. Cells were grown in DCCM media for 24 h followed by sialic acid supplementation (5 mM) for another 24 h. TRITC-phalloidin staining was done and images were captured at 555 nm at 60×. Statistical analysis was conducted using one-way ANOVA test. *p*-value for * is <0.05, ** is <0.005, and *** is <0.0005.

In order to validate the reduced level of F-actin in GNE mutant cells, actin filaments were stained using TRITC-phalloidin after 24-h starvation in DCCM media as mentioned in *Materials and Methods*. The quantitative analysis of F-actin levels by FluoViewFV1000 ver1.7a software demonstrated 50% reduction in TRITC-phalloidin intensity of D207V-GNE and V603L-GNE mutant cells as compared to wild-type GNE and vector control cells (Figure 2A). Consistent with the above results, fibronectin stimulation and sialic acid supplementation restored the intensity of TRITC-phalloidin in GNE mutant cells similar to wild-type and vector control cells (Figures 2B,C). Thus, our study clearly demonstrated that F-actin levels were drastically reduced in cells overexpressing GNE mutant proteins, which could be rescued after sialic acid supplementation. Also, the sialic acid-deficient cells were responsive to fibronectin stimulation leading to actin polymerization and shifting F/G-actin ratios.

### Effect of GNE on Actin Polymerization and Actin-Binding Partners

Since levels of F/G-actin ratios were altered in the presence of GNE mutant proteins, it was of interest to determine whether GNE has a role in actin polymerization per se. To address this, *in vitro* actin polymerization was conducted using rabbit skeletal actin and pyrene-tagged actin. Rabbit skeletal actin and Pyrene-tagged actin were mixed in the ratio of 9:1 and act as a source of G-actin, and polymerization was induced using KCl (as mentioned in *Materials and Methods*) in the presence of lysates isolated from GNE mutant cells and control cells that provide the cellular condition.

**Figure 3:**
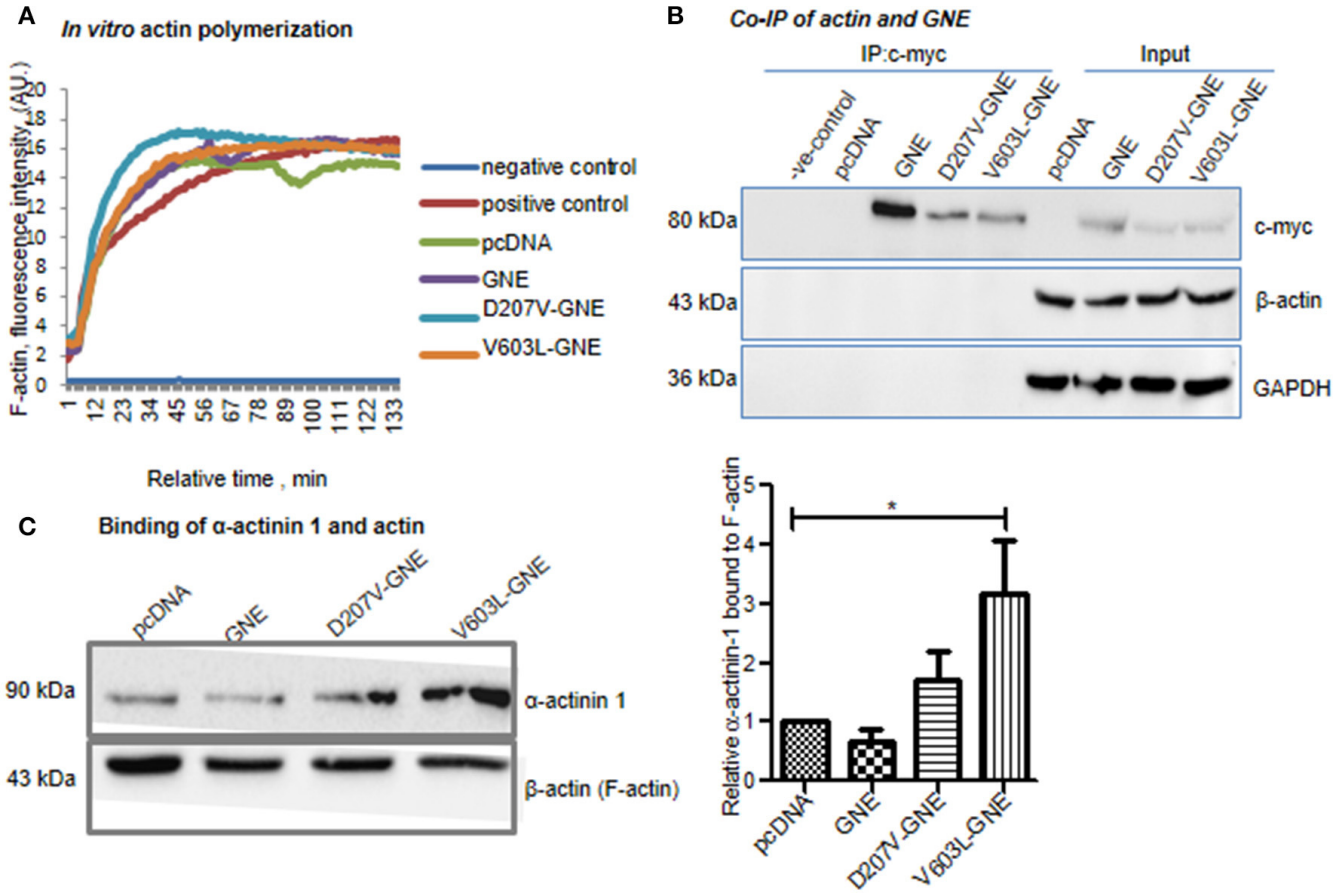
Effect of mutation in GNE in actin polymerization. **(A)** *In vitro* actin polymerization assay of GNE mutant cells and control. The assay was conducted using rabbit skeletal muscle actin and pyrene-tagged actin. HEK293 cells with overexpressed GNE mutant cells, wild-type GNE, and vector control cells were grown in DCCM media, and protein lysates were prepared in F-actin stabilizing buffer. The protein lysates (100 μg) were added to the *in vitro* actin polymerization assay reaction mixture. The polymerization of actin was checked at 350 nm/407 nm (excitation/emission) for 130 min recorded for an interval of 15 s. **(B)** Co-immunoprecipitation of GNE and actin (β-actin). Cells were lysed with RIPA buffer and 1 mg of protein lysates was incubated for immunoprecipitation with anti-c-Myc antibody after pre-clearing the lysate followed by incubation with A/G agarose beads. The immunoprecipitated beads were washed and bound proteins were released by boiling at 100°C for 10 min. The co-immunoprecipitation of GNE and actin was checked by immunoblotting with anti-β-actin antibody. **(C)** Effect of GNE mutation on α-actinin-1 binding with F-actin. F-actin fractions were isolated, and samples were loaded in SDS-PAGE followed by immunoblotting with anti-α-actinin-1 antibody and anti-β-actin antibody. The graph represents the relative α-actinin-1 bound to F-actin fraction analyzed using ImageJ software. The statistical analysis was conducted using one-way ANOVA test. *p*-value for * is <0.04.

The fluorescence was measured at 350/407 nm (excitation/emission) with an interval of 15 s for 130 min. The kinetic data plotted (Figure 3A) showed that there was no significant change in pattern of actin polymerization in GNE mutant cells D207V-GNE and V603L-GNE as compared to wild-type GNE and vector control cells. This observation indicates that GNE does not directly affect the actin polymerization *in vitro*. Thus, the question remains as to how a mutation in GNE affected the actin levels. Recent reports suggested that GNE interacts with actin-binding proteins α-actinin-1 and α-actinin-2 (Amsili et al., 2008; Pogoryelova et al., 2018). However, the binding of actin to GNE is not studied yet. Here, we performed co-immunoprecipitation of Myc-tagged GNE using anti-Myc antibody in GNE mutant cells and the control cells to address whether GNE binds directly to actin protein. The immunoprecipitated complex was immunoblotted with anti-β-actin antibody to check the binding of GNE with actin. As shown in Figure 3B, the right size bands of actin (43 kDa) and GNE (79 kDa) were observed in the flow-through fraction. However, no binding of actin with GNE could be observed in these blots, suggesting that GNE does not bind directly with actin in the cell.

GNE was reported to bind to α-actinin-1 and−2 (actin filament crosslinker) (Amsili et al., 2008; Harazi et al., 2017). Furthermore, binding of α-actinins to actin regulates the filament length as reported previously (Biron and Moses, 2004; Kemp and Brieher, 2018). We addressed whether binding of GNE to α-actinin-1 modulates the binding affinity of α-actinin-1 to actin filament and how it affects the F-actin formation in GNE mutant cells. To elucidate the effect of mutation in GNE on α-actinin-1 and F-actin binding property, we fractionated the F-actin fraction from the cell lysate and 40 μg of F-actin fractions was loaded on SDS-PAGE followed by immunoblotting with α-actinin-1 antibody. Densitometric analysis of the immunoblots with α-actinin-1 and β-actin antibody showed two-fold and three-fold increase in the F-actin-bound α-actinin-1 in the two mutants D207V-GNE (SEM ±1.3) and V603L-GNE (SEM ±3.3), respectively, as compared to wild-type GNE and vector controls (Figure 3C). Previous reports indicated that binding of GNE to α-actinin-2 increased 10-fold in GNE mutant (M743T-GNE) cells as compared to normal cells (Harazi et al., 2017). Our finding suggests that mutation in GNE affects the function of α-actinin-1 and its binding property to F-actin.

### Involvement of GNE in the Focal Adhesion Complex

**Figure 4:**
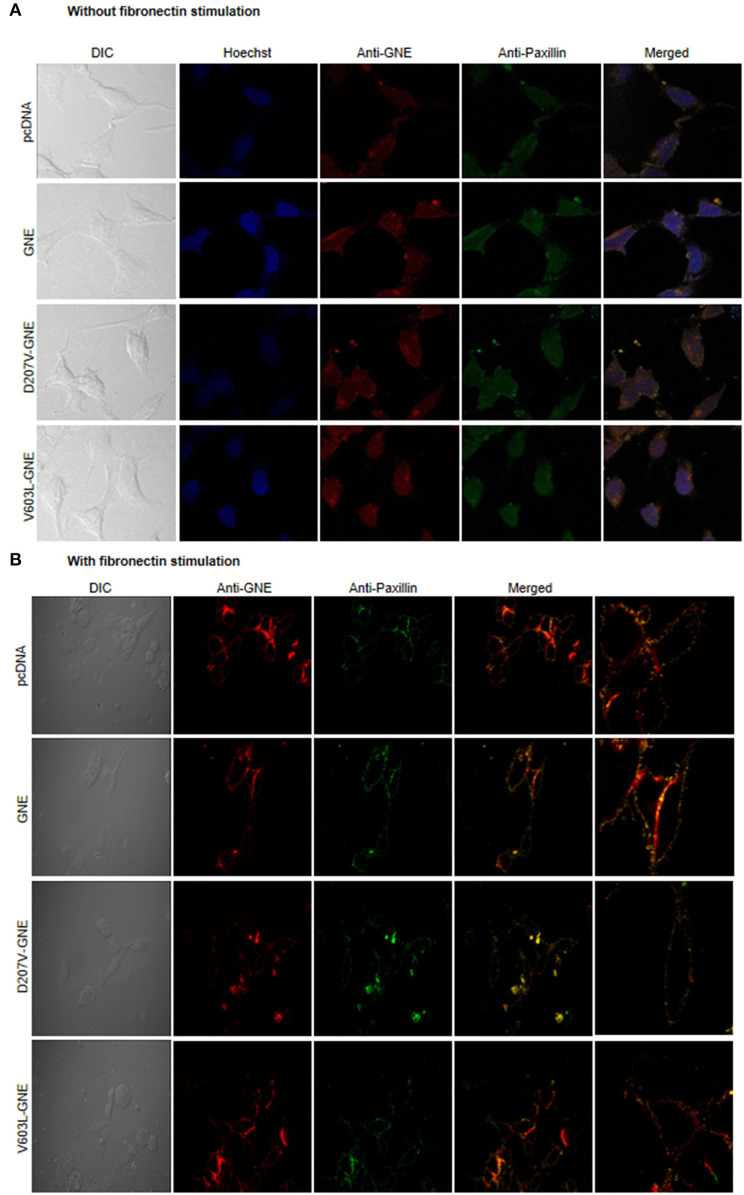
Localization of GNE on focal adhesion point. **(A)** HEK293 cells with overexpressed GNE mutant cells and control cells were grown in DCCM media and immunostained with anti-GNE and anti-Paxillin antibodies followed by counterstaining with Alexa Fluor 555 (GNE-red) and Alexa Fluor 488 (Paxillin-green). Images were captured at 60× magnification in confocal microscope. **(B)** Cells were stimulated with fibronectin for 4 h after growing in DCCM media for 24 h. Fibronectin-stimulated cells were fixed with paraformaldehyde and immunostained with anti-GNE and anti-Paxillin antibodies followed by counterstaining with Alexa Fluor 555 (GNE-red) and Alexa Fluor 488 (Paxillin-green). Images were captured at 60× magnification in a confocal microscope.

Integrins in their resting state, the cytoplasmic tail of α and β integrin, remain bonded to each other, forming a bent structure (Shattil et al., 2010; Anthis and Campbell, 2011). However, the binding of ectodomain of integrin with ECM results in a conformational change and recruits different adaptor proteins including FAK, Src, Paxillin, kindlin, talin, and α-actinins that form focal adhesion assembly to further regulate the actin dynamics (Parsons et al., 2010). Paxillin has an important role in the assembly or disassembly of the focal adhesion complexes as it controls various intracellular responses due to interaction of integrin with the extracellular matrix and, thus, regulates the organization of the actin cytoskeleton and migration of the cells (López-Colomé et al., 2017). Since GNE binds to α-actinin-1 and α-actinin-2, it is of interest to determine whether GNE is localized in focal adhesion assembly and involved in integrin signaling. For this purpose, GNE mutant cells were immunostained with anti-GNE and anti-Paxillin antibodies to study co-localization of proteins in focal adhesions using confocal microscopy. In the absence of fibronectin stimulation, no focal adhesions were observed in the membrane (Figure 4A). However, after fibronectin stimulation, GNE was found to be localized in the focal adhesion complex along with Paxillin (Figure 4B). This study indicates that GNE localizes in the focal adhesion complex after fibronectin stimulation and may play a role in integrin signaling pathway.

### Effect of GNE on RhoA Signaling Protein

**Figure 5:**
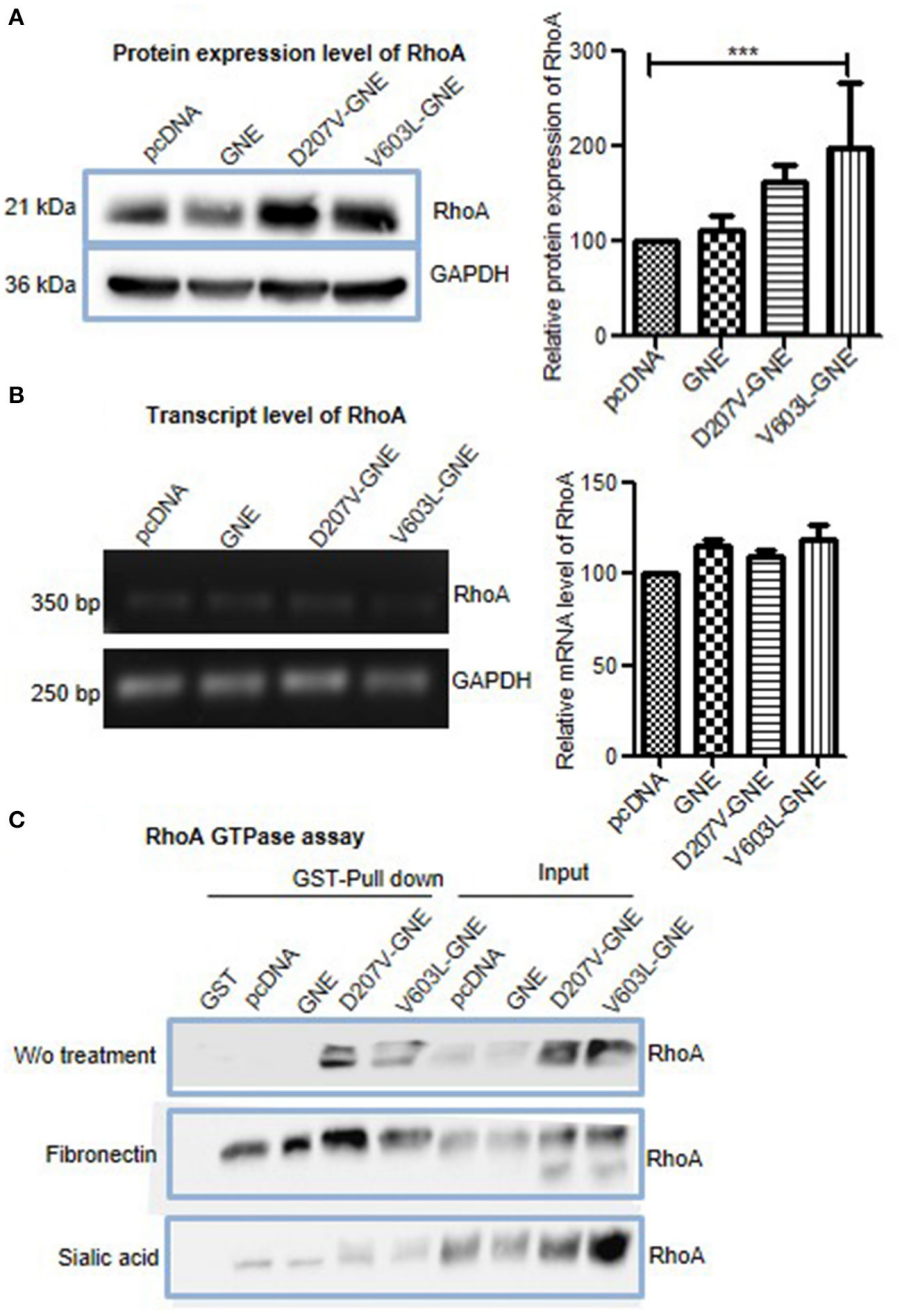
Effect of mutations in GNE on RhoA. **(A)** HEK293 cells with overexpressed GNE mutation (D207V-GNE and V603L-GNE) and control cells were grown in DCCM medium for 24 h and lysed using RIPA buffer. Fifty micrograms of proteins was loaded on SDS-PAGE followed by immunoblotting with anti-RhoA antibody. The graph represents the densitometry analysis of the blot using ImageJ software, and statistical significance of the data was assessed by one-way ANOVA test. *p*-value for *** is <0.0001 and the bar represents SEM, *n* = 3. **(B)** Transcript-level analysis of RhoA. RNA was isolated using TRIZOL reagent, and cDNA synthesis was done using RT-PCR. Semiquantitative qRT-PCR using specific primer for RhoA was done. The graph represents the densitometry analysis of the blot using ImageJ software. **(C)** Rho GTPase assay for the study of RhoA activity in GNE mutant cell. HEK293 cells with overexpressed GNE mutation (D207V-GNE and V603L-GNE) and control cells were grown in DCCM media for 24 h and lysed with GST-FISH buffer, and 1 mg of protein lysate was incubated with GST-RBD-bound Sepharose beads. The beads were washed with PBS and bound RhoA was dissociated by boiling for 10 min. The GTP-bound RhoA was detected by immunoblotting using anti-RhoA antibody.

The activation of focal adhesion by integrins triggers activation of small G proteins Rho/Rac/CDC42. RhoA acts as a switch regulating different actin-binding proteins in response to fibronectin-mediated integrin signaling (Kutys and Yamada, 2015). Therefore, we further assessed the effect of GNE mutation on RhoA GTPase function. First, the levels of RhoA protein in D207V-GNE and V603L-GNE mutant cells were determined by immunoblotting in D207V-GNE and V603L-GNE mutant cells. As shown in Figure 5A, RhoA protein expression levels were increased in GNE mutant cells compared to GNE wild-type and vector control cells suggesting RhoA upregulation. We further elucidated whether the increased RhoA protein levels were due to altered transcriptional regulation. The qRT-PCR using specific primers was performed from mRNA isolated from the GNE mutant cells (D207V-GNE and V603L-GNE) and control cells. The densitometric analysis of PCR products run on agarose gel reveal no change in the mRNA level of RhoA in the GNE mutant cell and control cells (Figure 5B). This study suggests that RhoA protein levels are upregulated in sialic acid-deficient cells without having an effect on its transcription.

RhoA function is regulated by its GTPase activity that converts active GTP-RhoA to inactive GDP-RhoA (Marjoram et al., 2014). The levels of GTP-bound RhoA were determined by GST pull down assay where the GST-tagged RhoA binding domain (GST-RBD) pulled the activated form of RhoA. Purified GST-RBD protein was incubated with cell lysates from GNE mutant cells to isolate active GTP-RhoA. As shown in Figure 5C, increased levels of GTP-RhoA were observed in GNE mutant cells compared to control cells. Upon fibronectin stimulation, increased RhoA activation was observed in GNE mutant cells, which is indicative of active integrin signaling pathway (Figure 5C). Also, sialic acid supplementation showed activated RhoA levels in GNE mutant cells similar to control cells (Figure 5C). Our study indicates increased activation of RhoA in GNE mutant cells that may be responsible for alterations in actin dynamics observed in sialic acid-deficient cells.

### Effect of GNE on Cofilin, F-Actin Severing Protein

**Figure 6:**
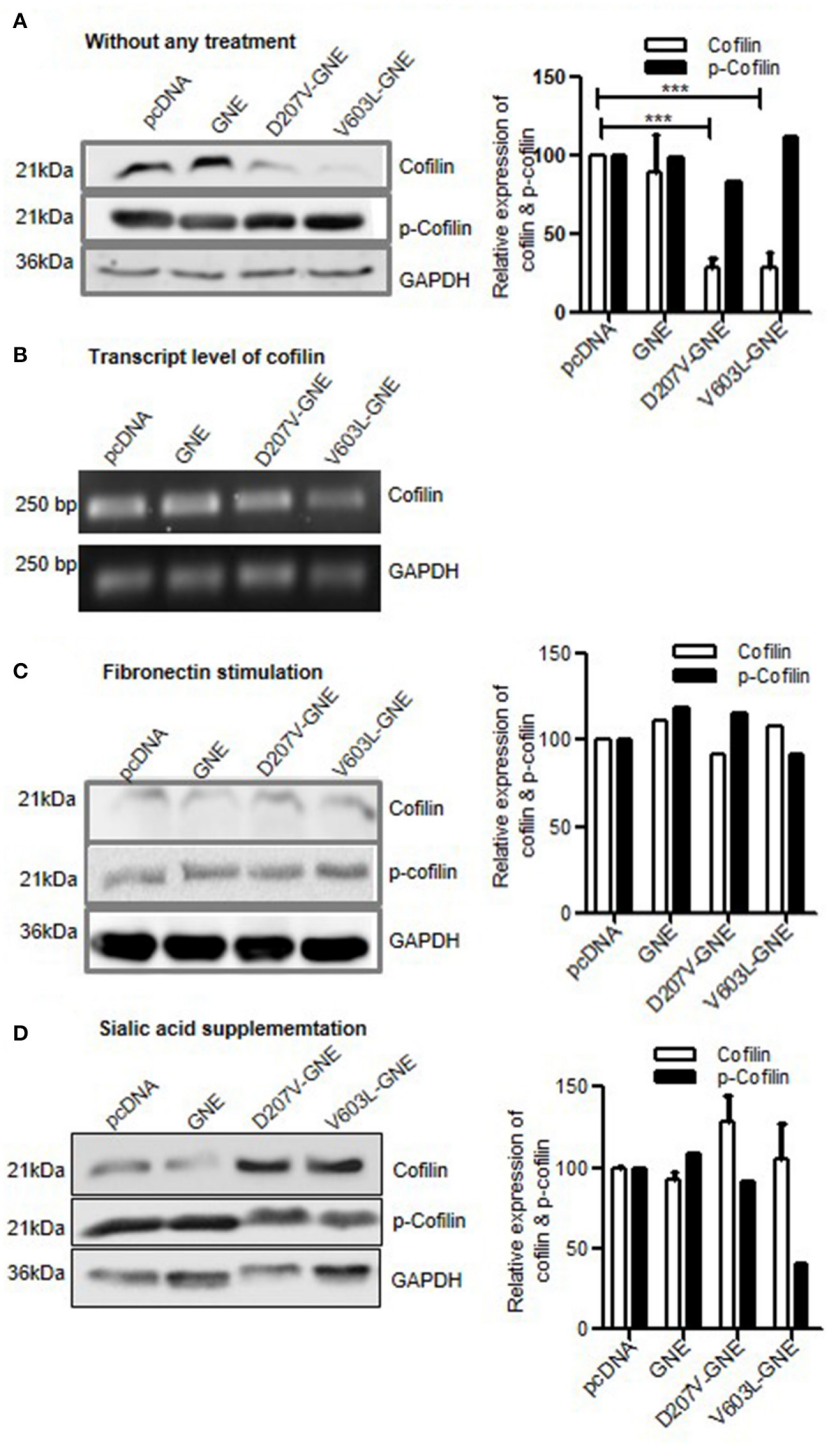
Effect of GNE mutation on Cofilin activity. **(A)** HEK293 cells with overexpressed GNE mutations and control cells were grown in DCCM media for 24 h, and protein lysate was prepared using RIPA buffer. Protein expressions were checked using SDS-PAGE separation followed by immunoblotting using anti-Cofilin and anti-phospho-Cofilin antibody. Densitometric analysis was conducted using ImageJ, and statistical significance was assessed with ANOVA test and *p*-value for *** is 0.0003. **(B)** Transcript-level analysis of Cofilin. RNA was isolated using TRIZOL reagent and cDNA synthesis was done using RT-PCR. Semiquantitative qRT-PCR using specific primer for Cofilin was done. **(C)** HEK293 cells with overexpressed GNE mutations and control were grown in DCCM media for 24 h followed by fibronectin stimulation for 4 h. The protein lysates were prepared using RIPA buffer, and protein expressions were checked using SDS-PAGE separation followed by immunoblotting using anti-Cofilin and anti-phospho-Cofilin antibody. **(D)** Immunoblot for Cofilin and phospho-Cofilin using HEK293 cells with overexpressed GNE mutations and control after sialic acid supplementation.

Downstream effect of RhoA activation leads to phosphorylation of Cofilin by LIMK. The phosphorylation of Cofilin inactivates Cofilin, thereby inhibiting F-actin severing and depolymerization (Kanellos and Frame, 2016). In order to elucidate the direct role of RhoA in actin dynamics of sialic acid-deficient cells, we studied the expression and activation of Cofilin in GNE mutant cells. The immunoblot analysis of cell lysates from GNE mutant cells using anti-Cofilin antibodies revealed 50% reduction in Cofilin protein expression compared to control cells (Figure 6A). The transcript analysis using qRT-PCR showed 20% reduction in Cofilin mRNA levels of GNE mutant cells compared to control cells (Figure 6B). Our study indicates reduced Cofilin levels in sialic acid-deficient cells.

The effect of Cofilin downregulation on its activity was elucidated by deciphering the phosphorylation status of Cofilin. From the immunoblot analysis with phospho-Cofilin antibody, we observed slight elevated levels of phosphorylated Cofilin in the mutant cells (D207V-GNE and V603L-GNE) as compared to wild-type GNE and vector control (Figure 6A). This study indicates reduced expression and inactivation of Cofilin in the GNE mutant cells. Reduced Cofilin activity is indicative of its direct effect on actin turnover rate that may limit actin polymerization.

Upon fibronectin stimulation, both Cofilin and p-Cofilin (phosphorylated Cofilin) levels were restored in GNE mutant cells, which is indicative of active integrin signaling and actin dynamics (Figure 6C). Sialic acid supplementation restored Cofilin levels in GNE mutant cells but p-Cofilin levels were low (Figure 6D), which suggest that Cofilin is active to mediate F-actin severing and actin turnover. Our study indicates that hyposialylation status in the cell can affect Cofilin function downstream of RhoA to alter actin composition in the cell.

### Effect of Rhosin and CGA on Actin Dynamics

**Figure 7:**
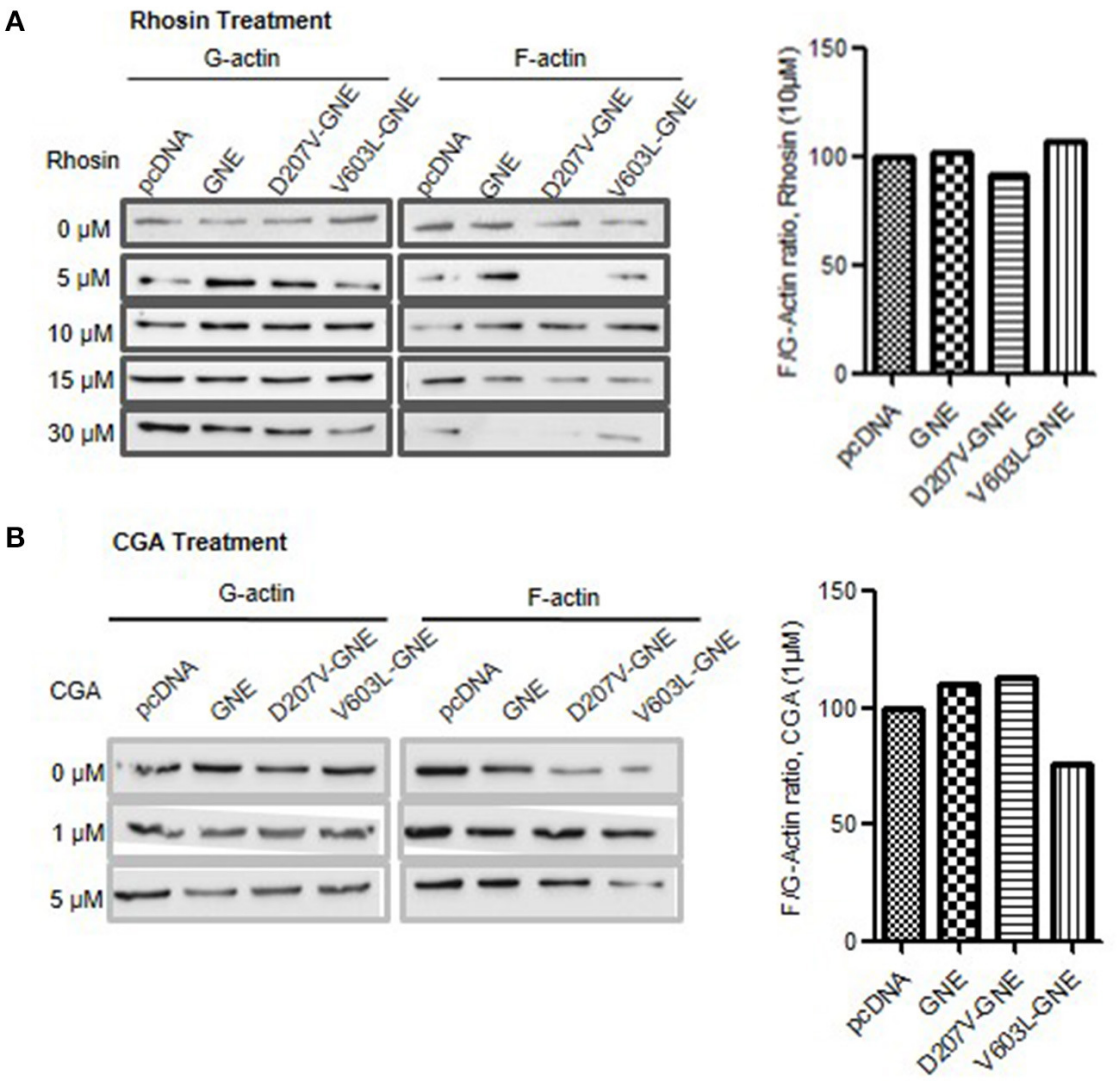
Effect of Rhosin and CGA on F/G-actin ratio in GNE mutant cells. **(A)** HEK293 cells with overexpressed GNE mutant cells along with the control cells were grown in DCCM media for 24 h followed by treatment of the cells with Rhosin in differential concentration (0, 5, 10, 15, and 30 μM). Cells were lysed in F-actin stabilizing buffer, and F- and G-actin fraction were separated. F-actin and G-actin fraction were loaded on SDS-PAGE and immunoblotting was done with anti-β-actin antibody. The graph represents densitometric analysis of the F/G-actin ratio for GNE mutant cells after 10 μM Rhosin treatment. **(B)** HEK293 cells with overexpressed GNE mutant cells along with the control cells were grown in DCCM media for 24 h followed by treatment of the cells with Rhosin at different concentrations (0, 1, and 5 μM). Cells were lysed in F-actin stabilizing buffer, and F-actin and G-actin fraction were separated. F-actin and G-actin fraction were loaded on SDS-PAGE, and immunoblotting was done with anti-β-actin antibody. The graph represents densitometry analysis of the F/G-actin ratio for GNE mutant cells after 1 μM CGA treatment.

In an attempt to identify other molecules besides sialic acid that may rescue defects in actin dynamics due to reduced sialic acid content and GNE mutation, we explored the modulators of RhoA and Cofilin proteins. Since RhoA was upregulated and Cofilin was downregulated in GNE mutant cells, we hypothesized that compounds inhibiting RhoA and activating Cofilin may contribute to regulate actin dynamics in these cells. To address this, Rhosin (an inhibitor of RhoA) treatment to GNE mutant cells followed by F/G-actin ratio analysis was done. As shown in Figure 7A, restoration of F/G-actin ratio in GNE mutant cells was observed at 10 μM concentration. We observed that lower concentration of Rhosin was ineffective while the higher concentration of Rhosin had negative effect in these cells.

In order to activate Cofilin, it needs to be dephosphorylated by phosphatases such as PP2A or PP2B. CGA is a compound that activates PP2B by dephosphorylating Cofilin at Ser-3. Therefore, GNE mutant cells were treated with CGA (1 and 5 μM) to determine the effect on F/G-actin ratio in these cells. As shown in Figure 7B, treatment of GNE mutant cells with 1 μM CGA restored F/G-actin ratios similar to GNE wild-type and control cells. Our study suggests that RhoA and Cofilin modulators can restore the F-actin levels in GNE mutant cells and may prove to be useful in disseminating cytoskeletal defects due to GNE mutation.

### Effect of GNE on Cell Migration

**Figure 8:**
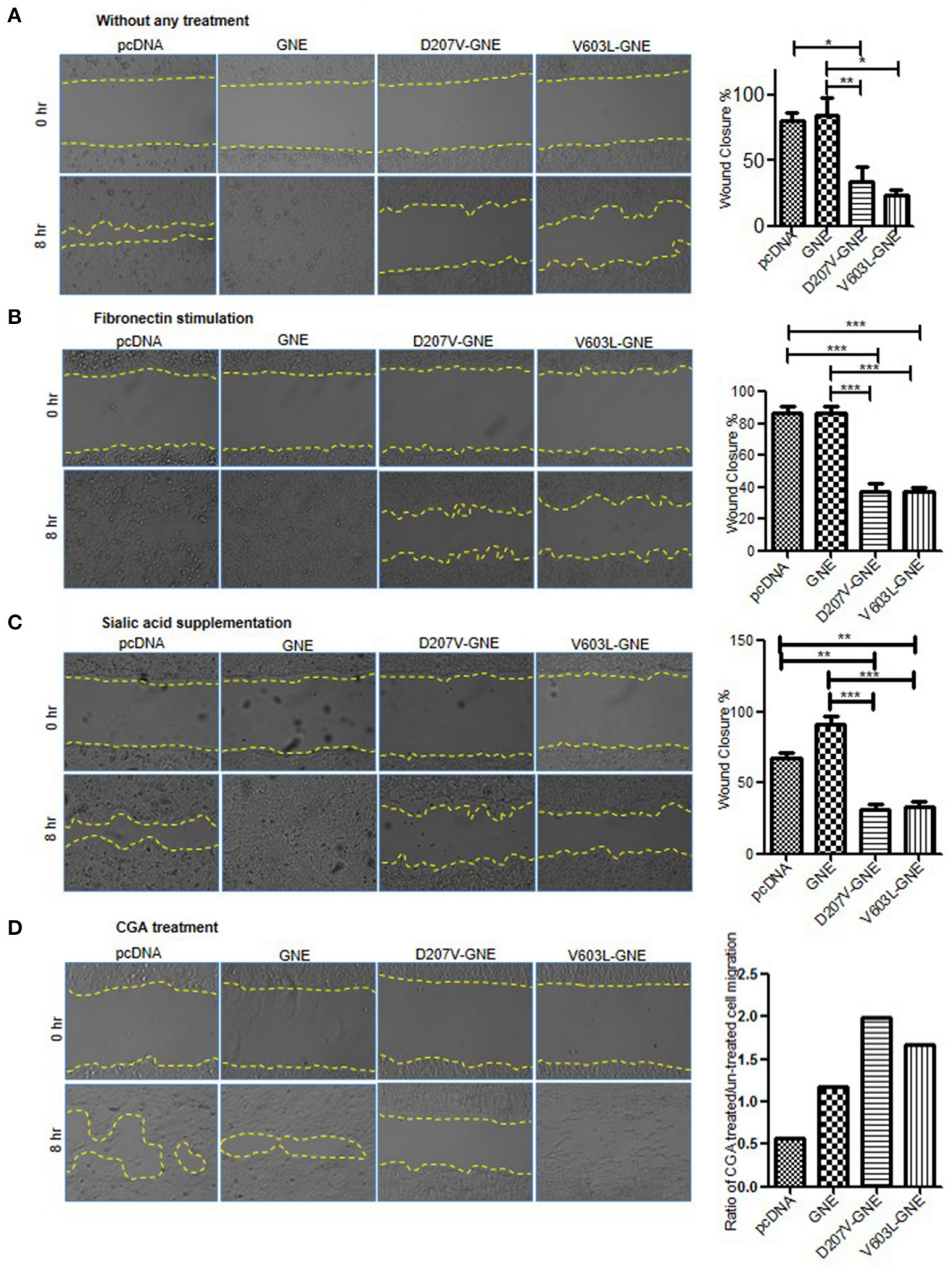
Wound healing assay of GNE mutant cells as compared to wild-type GNE and vector control. **(A)** HEK293 cells with overexpressed GNE mutation and control cells were grown in DCCM media for 24 h. Wound was created using IBIDI cell culture insert and images were captured at 20 × magnifications at 0 and 8 h in an inverted microscope. Percentage wound closure was calculated from area uncovered by cells using ImageJ software. Statistical significance was assessed with ANOVA test and *p*-value for * is 0.03 and ** is 0.003. **(B)** Wound healing assay of GNE mutant cells and control cell after fibronectin stimulation. Statistical significance was assessed with ANOVA test and *p*-value for *** is 0.0001. **(C)** Wound healing assay of GNE mutant cells and control after sialic acid supplementation. Statistical significance was assessed with ANOVA test and *p*-value for ** is 0.003 and *** is 0.0001. **(D)** Wound healing assay of GNE mutant cells and control after 1 μM of CGA treatment.

The overall effect of changes in actin dynamics culminates to improper cell adhesion and migration. GNE mutant cells showed upregulation of RhoA, Cofilin deactivation, and reduced F/G-actin ratio leading to reduced actin polymerization. How these signaling changes affected the cell migration process was determined through wound healing assays. GNE mutant cells were wounded using IBIDI inserts to generate uniform cuts followed by cell imaging at regular intervals in an inverted microscope to determine the distance of migration. The GNE mutant cells showed approximately 40% reduction in wound closure activity (D207V-GNE; SEM ±3.7 and V603L-GNE; SEM ±4.5) compared to wild-type GNE and vector control (Figure 8A). In other words, GNE mutant cells migrated slowly compared to GNE wild-type and vector control cells. Upon fibronectin stimulation, GNE mutant cells showed approximately 50% reduced migration (D207V-GNE, SEM ±8.1 and V603L-GNE, SEM ±8.2) as compared to wild-type GNE and vector control (Figure 8B). These data support our earlier observation where GNE mutant cells showed increased adhesion in response to fibronectin stimulation. Also, sialic acid supplementation could not restore the cell migration activity of GNE mutant cells, indicating an alternate role of GNE in cell migration (Figure 8C). Interestingly, although treatment of GNE-deficient cells with Rhosin did not increase the cell migration property, CGA treatment significantly led to migration of GNE-deficient cells (Figure 8D). Approximately, 40% increase in D207V-GNE cell migration and 20% increase in V603L-GNE cell migration was observed compared to vector control after CGA treatment. Our study indicates that molecules regulating cytoskeletal organization such as actin via Cofilin can offer a therapeutic target for GNE-deficient cellular functions.

## Discussion

Cell migration and adhesion plays an important role in survival of the cell and help the cell to respond to external cues, immune cell functioning, growth and development, and differentiation (Guo et al., 2002; Suzuki et al., 2015; Läubli and Borsig, 2019). The general cell migration follows four basic steps—formation of protrusion also known as lamellipodium in the front in response to external cues and integrin activation, formation of focal adhesion leading to a strong bond between the ECM, retraction of the rear end from the ECM, and disassembly of focal adhesion complexes from the rear that detach the cell and allow the cell to move forward (Parsons et al., 2010). The formation of lamellipodia is controlled by the actin polymerization regulated through different actin-binding proteins like Arp2/3 and Cofilin (Swaney and Li, 2016). Recent studies indicate that cell surface sialylation affects cell adhesion and invasion to the extracellular matrix resulting in modulation of biological behavior (Guo et al., 2002; Suzuki et al., 2015; Läubli and Borsig, 2019). In particular, influence of sialic acid determinants on integrin signaling pathways altering cell-ECM migratory phenomenon has been shown in various pancreatic and gastric cancers (Bassagañas et al., 2014a,b). Downstream to integrin, various factors regulate signal transduction from ECM to cytoskeletal protein dynamics. Alterations in glycosylation of cell surface proteins as well as cytoskeletal proteins have been shown to affect cell adhesion and migration (Hsiao et al., 2017; Muniz de Queiroz et al., 2019).

A previous report from our laboratory demonstrated that defect in sialic acid biosynthetic pathway due to mutation in GNE led to hyposialylation of integrin and increased adhesion of cells to fibronectin (Grover and Arya, 2014). In the present study, effect of hyposialylation on integrin downstream signaling pathway involving RhoA and Cofilin was studied to understand actin dynamics and cell migration. The pathological significance of the study relates to understanding defects in cytoskeletal protein function observed in mutated GNE, which could have therapeutic implications in neuromuscular disorder, GNE myopathy, as well as certain cancers with altered sialic acid content (Läubli and Borsig, 2019).

Glycosylation of actin has been reported to regulate its dynamic levels as well as cardiac and skeletal muscle contraction (Hedou et al., 2007; Ramirez-Correa et al., 2008; Zhang et al., 2018). O-GlcNAcylation and glucose affect the formation of F-actin in different studies (Sulochana et al., 2001; Resmi et al., 2005). In this study, how alteration in cellular sialic acid content due to GNE mutation affected actin dynamics was addressed. Our results showed 55 and 40% reduction in F/G-actin ratio in GNE mutant cells (D207V-GNE and V603L-GNE, respectively), as compared to wild-type GNE and vector control cells. Even the TRITC-phalloidin staining showed a drastic reduction in F-actin levels of GNE mutant cells. Since, actin dynamic levels in the form of F-actin (filamentous/polymerized) and G-actin (globular/monomer) are central to regulate cytoskeletal organization in the cell, reduction in F-actin levels would affect G- and F-actin equilibrium. Alteration in G- and F-actin equilibrium would drastically affect the cross-talk between various proteins affecting cell adhesion and migration. Interestingly, another study showed that Aβ-accumulation mediates increased depolymerization of F-actin in the dendritic spine leading to cognitive impairment in Alzheimer's disease (Kommaddi et al., 2018). GNEM, a neuromuscular disorder, shows rimmed vacuole formation and protein aggregates composed of β-amyloid, Tau, and presenilin in muscle cells. Thus, reduced F-actin levels could also be a consequence of Aβ-accumulation in GNE mutant cells. This is the first report indicating reduced F-actin levels in sialic acid-deficient cells.

The hyposialylation of cell surface receptors such as integrin, NCAM, Neprilysin, transferrin, and α-dystroglycan caused abnormal cell function (Huizing et al., 2004; Ricci et al., 2006; Broccolini et al., 2008; Grover and Arya, 2014). Sialylation of integrin regulates its affinity toward ECM, thereby regulating adhesion and migration of the cell (Lee et al., 2010; Yuan et al., 2016). Sialic acid and its precursor have been in clinical trials for the treatment of GNEM, suggestive of restoring the defect due to GNE mutation. Therefore, it is of interest to determine the effect of sialic acid supplementation on GNE mutant cells toward F-actin levels. Restoration of F-actin levels after supplementation of sialic acid in GNE mutant cells as observed by immunoblots and TRITC-phalloidin staining suggest that sialic acid may restore loss of function due to GNE mutation such as integrin hyposialylation. Whether sialylation of integrin is sufficient to mediate downstream signaling events and cell adhesion/migration property, or GNE has an alternate role, needs to be explored further.

Assembly and disassembly of actin filament is a continuous process tightly regulated by a myriad of actin-binding proteins. No change in the *in vitro* actin polymerization activity of GNE mutant cells indicate that GNE has no role in actin polymerization directly; rather, changes in actin dynamic level could be an indirect consequence of mutation in GNE probably via Cofilin, profilin, Arp2/3, etc. Though GNE does not bind to actin directly in our study, Amsili reported GNE binding to α-actinin-1 and α-actinin-2, actin-binding proteins (Amsili et al., 2008; Kovac et al., 2013; Kemp and Brieher, 2018). Harazi et al. also showed that M743T mutant GNE binds α-actinin-2 10-fold more strongly than α-actinin-1 (Harazi et al., 2017). We observed increased binding between α-actinin-1 and F-actin in GNE mutant cells, D207V-GNE, and V603L-GNE as compared to wild-type GNE and vector control. Since tropomyosin stabilizes F-actin, strong binding of actinin to actin in GNE mutant cells could prevent tropomyosin binding to F-actin, thereby causing disassembly of F-actin units (Yu and Ono, 2006; Sui et al., 2017).

Integrin protein not only acts as anchoring the F-actin filament but also activates a large number of signaling molecule that regulates the actin dynamics. Focal adhesion proteins like Paxillin, Src, and FAK activate different GEFs and GAPs, which can regulate the activity of RhoGTPases. The co-localization of GNE along with Paxillin in the focal adhesion after fibronectin stimulation suggests the involvement of GNE in the integrin signaling. Among the different RhoGTPases, RhoA was reported to be important for focal adhesion maturation and regulation of stress fibers (Lawson and Burridge, 2014). The upregulation of RhoA in the GNE mutant cells at the protein level but not at the mRNA level indicates effects on RhoA protein function in GNE-deficient cells. Active RhoA directly regulates stress fiber formation by reducing F-actin filament formation (Katoh et al., 2011). We found increased GTP-bound RhoA in D207V-GNE and V603L-GNE compared to control cells indicating RhoA activation in GNE mutant cells that may contribute to reduced F-actin. Alteration in glycosylation of G-CSF had been shown to affect RhoA activation in human leukemic cells (Mattii et al., 2011). Sialic acid supplementation in GNE mutant cells showed increased RhoA activation levels compared to control cells, suggesting an indirect effect of GNE mutation on RhoA. Fibronectin stimulation triggering integrin signaling response led to RhoA activation in GNE mutant cells. Thus, RhoA activation in GNE mutant cells could be a consequence of mutation in GNE and molecules that alter RhoA function may restore cellular defect due to GNE mutation. Interestingly, treatment of GNE mutant cells with RhoA inhibitor Rhosin could restore the F/G-actin ratio and opens a new dimension to look for alternate targets in GNE mutant cells.

The downstream effector molecule for RhoA is ROCK, which activates LIMK. LIMK further phosphorylates Cofilin, the actin depolymerizing factor. As an actin severing protein, Cofilin plays a crucial role in dynamic reorganization of actin cytoskeleton and cell migration (DesMarais et al., 2004). Our study showed 50% reduction in Cofilin protein levels in GNE mutant cells compared to control cells, indicating downregulation of Cofilin protein expression. In addition to actin depolymerizer, Cofilin has been shown to promote actin polymerization that mediates direction of cell motility (Ghosh et al., 2004). Thus, reduced Cofilin levels in GNE mutant cells could affect actin polymerization and contribute to reduced F-actin filament levels. Further, Cofilin is regulated by O-GlcNacylation at Ser-108 and phosphorylation at Ser-3. Loss of Cofilin O-GlcNacylation impairs cell invasion (Huang et al., 2013), while Cofilin phosphorylation inactivates Cofilin, thereby hampering actin depolymerization (Sidani et al., 2007; Chung et al., 2013). We found Cofilin to be phosphorylated and that the p-Cofilin/Cofilin ratio is high in GNE mutant cells, which would inhibit actin severing and recycling of G-actin monomers in the cytoplasmic pool. Cofilin phosphorylation and inactivation was shown to accelerate the aging process in Alzheimer's pathology and contribute to cognitive function (Barone et al., 2014). Reduction in Cofilin activity of two Nemaline myopathy patients resulted in reduced actin depolymerization and muscle weakness (Agrawal et al., 2006). Therefore, downregulation and inactivation of Cofilin in GNE mutant cells could significantly affect actin function and cytoskeletal organization in GNE mutant cells. Sialic acid supplementation restored Cofilin expression levels and p-Cofilin/Cofilin ratio reduced, indicative of Cofilin activation and increased F-actin levels. Also, fibronectin stimulation triggering integrin signaling response showed reduced p-Cofilin/Cofilin ratio, indicative of Cofilin inactivation required for actin polymerization and cell migration. Interestingly, small molecules such as CGA activate phosphatase PP2B to dephosphorylate Cofilin (Wang et al., 2005). Treatment of GNE mutant cells with CGA resulted in dephosphorylation of Cofilin, thereby activating Cofilin and restoring F-actin levels in GNE mutant cells. Thus, our study indicates that Cofilin can play a very important role in cytoskeletal organization of GNE-deficient cells. Molecules that alter Cofilin function can counterbalance the cellular defects due to GNE mutation and be potential therapeutic targets.

Any alteration in the actin dynamic level of cells is reflected in cell migration as actin provides force to move the cells (Fournier et al., 2010; Cramer, 2013). Reduction in F-actin levels of GNE mutant cells correlated with reduction in migration of GNE mutant cells as measured by wound healing assays. Fibronectin stimulation also showed 10% reduction in cell migration of GNE mutant cells compared to control cells, indicating that hyposialylation affected the cell migration property of the cell. However, supplementation with sialic acid did not restore cell migration property of GNE mutant cells while treatment with CGA improved cell migration capacity of D207V-GNE mutant by 40% and V603L-GNE mutant by 20%. This suggests that small molecules affecting cytoskeleton proteins such as Cofilin directly can be effective in rescuing the cellular defects due to mutation in GNE and offer promising therapeutic target for drug design. The study also supports an alternate role of GNE in cell migration as sialic acid is not sufficient to restore the phenotype. GNE may interact with focal adhesion assembly via actinin to affect Src and FAK signaling, leading to RhoA activation. GNE was also shown to interact with HSP70, which regulates RhoA activation and protein aggregation (unpublished observation). The phenomenon of apoptosis observed in GNE mutant cells and reduced F-actin levels may contribute to cell shrinkage and loss of cell shape (rounding off). All these studies indicate that GNE may play an alternate role in regulating cellular functions.

For many years, treatment of GNE myopathy has been focused on restoration of sialylation level and many attempts had been made to treat GNE myopathy by supplementing sialic acid and its derivatives—ManNAc, Aceneuraminic acid-ER, etc. Unfortunately, all the attempts made using sialic acid derivatives failed to cure the disease. Our present study shows that GNE protein, apart from being the critical enzyme in sialic acid biosynthesis, is involved in regulation of actin dynamics in the cell that in turn regulates cell migration. Even though sialic acid is very crucial for cell functioning, therapeutic treatment of GNE myopathy cannot be fulfilled just by restoration of sialylation as GNE is involved in signaling that modulates cell migration. Combinatorial drugs that support alternate GNE functions such as CGA for Cofilin may prove to be promising in rescuing defects due to GNE mutations. These studies need to be further validated in muscle cells or appropriate animal models for GNE myopathy.

### Proposed Model

**Figure 9:**
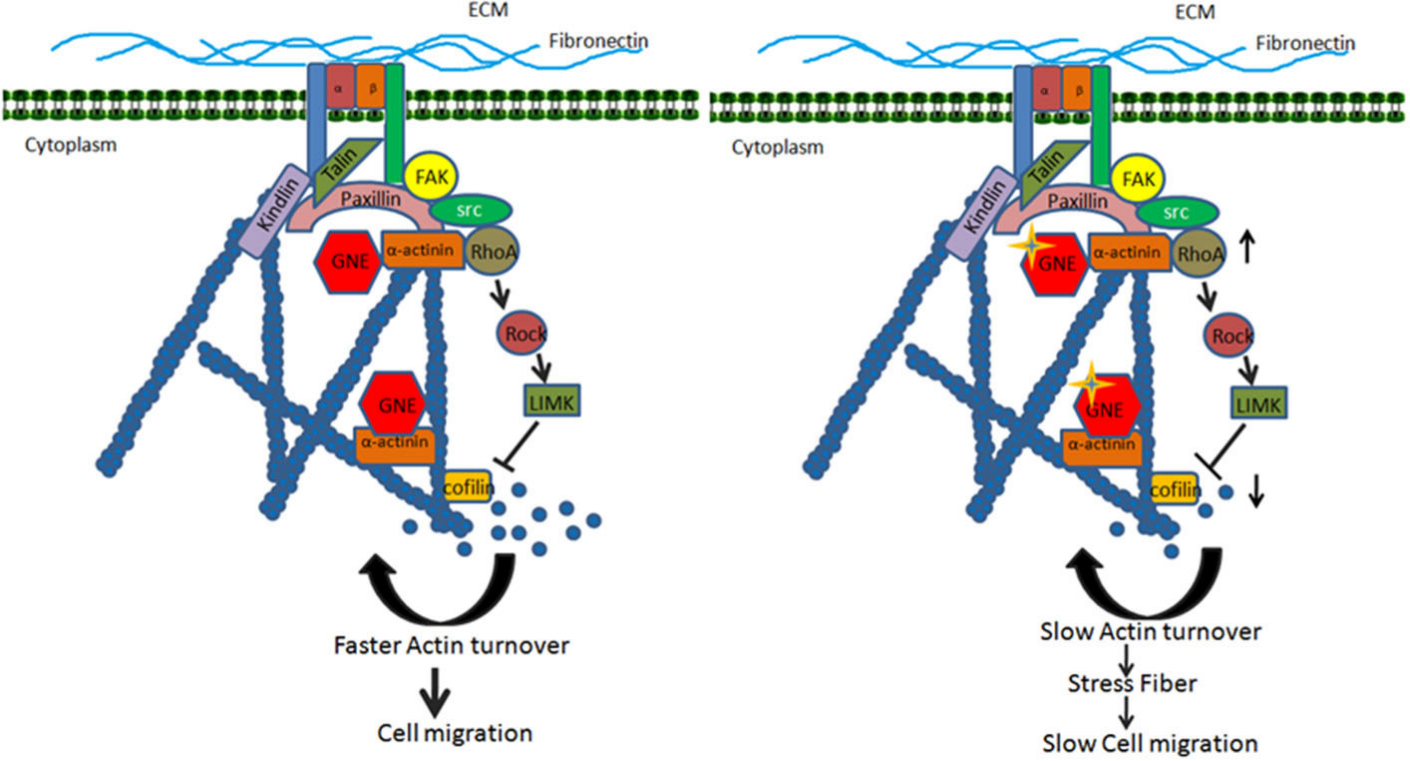
Proposed model for effect of GNE mutation on actin dynamics and cell migration. The figure shows that GNE is involved in integrin signaling pathway leading to regulation of cell migration. GNE interacts with α-actinin and localized in the focal adhesion complex where it regulates the activation of RhoA. RhoA activates its downstream signaling molecule leading to regulation of Cofilin, the actin severing molecule. The active turnover of actin filament by Cofilin fuels the G-actin pool required for actin polymerization and cell migration. In GNE mutant cells, the activity of RhoA is upregulated, which leads to downregulation of active Cofilin. The downregulation in Cofilin activity changes the dynamic of actin, resulting in low turnover of the actin and slow cell migration.

Based on the above findings, we proposed a model for the role of GNE in the cytoskeletal organization during cell migration (Figure 9). GNE is localized in the cytoplasm, Golgi, or nucleus in the cell to mediate sialic acid biosynthesis in the cell. In response to extracellular matrix stimulation such as fibronectin, GNE migrates to internal vesicles and membranes to localize in focal adhesions along with α-actinins, which are actin-binding proteins. GNE is reported to interact with α-actinin-1 and−2, with unclear function. The focal adhesion assembly takes place comprising of Src, paxillin, and FAK proteins that transduces the downstream signal leading to RhoA activation. Activated RhoA further activates LIMK, which phosphorylates Cofilin and inactivates Cofilin. Being an inhibitor of F-actin polymerization, reduced Cofilin activity supports actin polymerization. However, protein phosphatases such as PP2A and PP2B dephosphorylate Cofilin and activate it for actin severing function. This will generate G-actin monomers maintaining actin dynamics needed for cell migration. However, when GNE is mutated, it binds to α-actinins more strongly and forms focal adhesions upon fibronectin stimulation. In this situation α-actinins may bind more strongly to actin. The hyposialylated integrin binds strongly to the substratum and recruits GNE, FAK, Src, α-actinin, talin, kindlin, paxillin, etc. in focal adhesion complex. RhoA is further activated in GNE mutant cells leading to continuous increase in Cofilin phosphorylation and inhibition of Cofilin function. Due to the lack of Cofilin activity, actin severing is inhibited, and generation of G-actin monomer pool is inhibited. This slow turnover of actin causes stress fiber formation and reduced migration in GNE mutant cells. Thus, changes in actin levels as measured by phalloidin staining can be used as a readout for screening of compounds effective for GNE function.

## Data Availability Statement

The original contributions presented in the study are included in the article/supplementary material, further inquiries can be directed to the corresponding author/s.

## Author Contributions

RA conceptualized and design the study, analyzed and interpreted the data, and wrote the manuscript. She brought the funding to conduct the study. SD performed the experiments and compiled the data. She wrote first draft of the manuscript. RY validated the study by performing parallel studies. She helped in completing the study and edited the manuscript. All authors contributed to the article and approved the submitted version.

## Conflict of Interest

The authors declare that the research was conducted in the absence of any commercial or financial relationships that could be construed as a potential conflict of interest.

## Abbreviations

GNE: UDP N-acetylneuraminic 2-epimerase/N-acetylmannosamine kinase
F-actin: filamentous actin
G-actin: globular actin
FAK: focal adhesion kinases
Src: sarcoma
ECM: extracellular matrix
Arp 2/3: actin-related proteins 2/3
NCAM: neural cell adhesion molecule
GTP: Guanine Triphosphate
WASP: Wiskott–Aldrich syndrome protein
Rho: Ras homology family member A
Rac: Ras-related C3 botulinum toxin substrate
Cdc42: Cell division control protein 42
LIMK: LIM domain kinase
SSH1: Slingshot homolog 1
SUMO: Small Ubiquitin-like modifier proteins
IGF1R: Insulin growth factor 1 receptor
DMEM: Dulbecco's Modified Eagle Medium
RIPA: Radioimmunoprecipitation assay buffer
ECL: Enhanced Chemiluminescence
DABCO: 1,4-diazabicyclo [2.2.2] octane
TRITC: Tetramethyrhodamine
GST-FISH: Glutathione S-transferases FISH
PMSF: Phenylmethylsulfonyl Fluoride
RBD-GST: Rho Binding Domain-Glutathione S-transferases
GAPDH: Glyceraldehyde 3-phosphate dehydrogenase
HRP: Horseradish Peroxidase
PP2A: Protein phosphatase 2
PP2B: Serine/threonine-protein phosphatase 2B
CGA: Chlorogenic acid
GNEM: GNE myopathy
ROCK: Rho Associated protein kinase.
